# Infant feeding counseling and knowledge are the key determinants of prelacteal feeding among HIV exposed infants attending public hospitals in Ethiopia

**DOI:** 10.1186/s13690-017-0191-y

**Published:** 2017-05-22

**Authors:** Melaku Kindie Yenit, Haregewoyn Genetu, Amare Tariku

**Affiliations:** 1 Department of Epidemiology and Biostatistics, University of Gondar, Gondar, Ethiopia; 2 Gondar University Hospital, Gondar, Ethiopia; 3 Department of Human Nutrition, University of Gondar, Gondar, Ethiopia

**Keywords:** Prelacteal feeding, Factors, HIV positive mothers, Northwest Ethiopia

## Abstract

**Background:**

Despite the fact that breastfeeding promotes optimal health and growth for infants and young children, inappropriate feeding practice, such as prelacteal feeding increases the risk of neonatal death and illness and remains a public health problem in Ethiopia. Therefore, this study assessed the prevalence of prelacteal feeding and associated factors among HIV positive mothers with children aged 7–20 months attending government hospitals in North Gondar zone, northwest Ethiopia.

**Methods:**

An institution based cross-sectional study was conducted from February to March, 2016, at public hospitals of North Gondar Zone. Three hundred sixty-seven HIV positive mothers attending PMTCT clinics in government hospitals during the study period were included in the study. A multivariate logistic regression analysis was used to investigate factors associated with prelacteal feeding. The Adjusted Odds Ratio (AOR) with the corresponding 95% Confidence Interval (CI) was used to show the strength of association, and variables with a *P*-value of <0.05 were considered statistically significant.

**Results:**

In this study, the overall prevalence of prelacteal feeding was 19.1% (95% CI: 15–23). According to the multivariate analysis, prelacteal feeding was associated with fathers with no formal education (AOR = 5.85; 95% CI: 2.02, 16.92), lack of infant feeding counseling (AOR = 3.36; 95% CI: 1.27, 8.85), discarding the colostrum (AOR = 5.16; 95% CI: 2.32, 11.45), inadequacy of antenatal care visit (AOR = 0.07; 95% CI: 0.03, 0.15), and high IYCF knowledge (AOR = 0.10; 95% CI: 0.03, 0.30).

**Conclusion:**

In this study, the prevalence of prelacteal feeding was high. Furthermore, father’s education, colostrum feeding, infant feeding counseling, ANC visit, and IYCF knowledge were significantly associated with prelacteal feeding. As a result, strengthening maternal health care utilization, breastfeeding counseling, and IYCF knowledge are essential to address the high burden of prelacteal feeding.

## Background

Appropriate and optimal feeding practices for infants and young children are essential for healthy growth and cognitive development [1–5]. Breastfeeding initiation in the first hours of birth increases the chance of getting the colostrum and its benefits [6, 7], mother-infant bond [8], as well as successful establishment of breastfeeding [9]. In addition, breastfeeding reduces risks of contracting infectious [10–12] and non-infectious diseases [13–16], which in turn save millions of children from death. Furthermore, it helps postnatal weight management and delayed menstruation, suggesting extra contraceptive importance [17]. Due to these facts, the World Health Organization (WHO) and the United Nations Children’s Fund (UNICEF) recommend that breastfeeding should start within an hour of delivery and that exclusive breastfeeding be maintained for the first six months of the infant’s life. Furthermore, safe, adequate, and appropriate complementary feeding should be initiated at the age of six [3, 4, 18].

In the era of HIV/AIDS pandemicity, the dilemma of vertical transmission of the HIV virus through breastfeeding has been controversial, and it was previously pointed out that early cessation of breastfeeding reduces HIV transmission. However, recent reports from low and middle income countries, especially in Sub-Saharan Africa countries, indicate that childhood mortality and morbidity due to malnutrition and diarrheal diseases exceeds HIV infection [19, 20]. Therefore, WHO recommends focusing on childhood survival than on the transmission of HIV. Considering these facts, the current WHO guideline recommends that all HIV exposed infants from known HIV positive mothers get breastfeeding only for the first six months and that the children be given solid or semi-solid complementary food in addition to continued breastfeeding from the age of 6 months until 12 months [4, 18, 21].

Even though early initiation of breastfeeding promotes health and increases the duration of breastfeeding [1, 3, 4, 7, 22, 23], inappropriate breastfeeding practice after delivery, such as providing food or liquids before the establishment of breast milk is commonly practiced. Since the lining of neonatal gut is immature, any food or drink offered before the establishment of breast milk (also known as prelacteal feeding) can easily introduce infections and subsequently leads to death [24]. Prelacteal feeding is defined as offering any food or drinks before the establishment of breast milk in the first three days of delivery [3].

Global reports on childhood feeding practice indicated that the highest rate of prelacteal feeding was reported in Asia [24, 25], Latin America [26], and Africa [27, 28]. In Asia, prelacteal feeding practice ranged from 54.0–93.9% [24, 25, 29]. In Africa, where HIV/AIDS disproportionally hit, the prevalence of prelacteal feeding ranged from 5.4–75.2% [27, 28, 30]. Various reports indicated that prelacteal feeding was affected by various factors, but was mainly associated with maternal and health service related characteristics. Lack of education [24], poor household wealth status [24], maternal age, lack of counseling on breastfeeding [29, 31, 32], poor infant breastfeeding knowledge [33, 34], inadequate antenatal care visit [24, 35], and discarding colostrum [2] were some of the factors significantly associated with prelacteal feeding.

Ethiopia has been implementing infant and young child feeding strategy and other nutrition programs to improve optimal child feeding practices, thereby reducing morbidity and mortality [30]. Moreover, breastfeeding is nearly universal but it continues as sub-optimal. According to the 2011 Ethiopian Demographic and Health Survey (EDHS) report, only half (51.5 & 52%, respectively) of the mothers initiated breastfeeding within an hour of delivery and exclusively breastfed [36]. However, inappropriate feeding practices, including prelacteal feeding are responsible for 24% of infant deaths [37]. About 27.1% of the mothers offered prelacteal feeds in Ethiopia. Though there are regional variations in prelacteal feeding practices which ranged from 10.4–72.5% [2, 36, 38–41], the highest rates were reported in Somali (72.5%) and Amhara (47.8%), regions [36]. Despite the fact that prelacteal feeding has a negative effect on child health, little is known about its magnitude among HIV positive mothers. Therefore, this study aimed to assess prelacteal feeding and associated factors among HIV positive mothers with children aged 7–20 months attending government hospitals in North Gondar Zone, northwest Ethiopia.

## Methods

### Study setting and design

An institution based cross-sectional study was conducted from February to March, 2016, at North Gondar Zone government hospitals, northwest Ethiopia. The zone has 126 health centers and 6 hospitals providing services to the community. Out of the six hospitals in the study area, only three, Mettema, Dabark, and Gondar University were providing anti-retroviral treatment (ART) and prevention of mother-to-child HIV transmission (PMTCT) services at the moment. A total of 392 HIV infected mothers with children aged 7–20 were registered for HIV care and support at the PMTCT clinics of the selected hospitals.

### Sample size and sampling procedure

All HIV positive mothers who had children aged 7–20 months and were attending the PMTCT clinics participated in the study. Mother-child pairs were recruited from the three government hospitals of North Gondar Zone. Sample size was calculated using the single proportion formula with the following assumptions: 38.8% expected prevalence of prelacteal feeding in Raya Kobo, Amhara Region [36], 95% level of confidence, 5% margin of error, and 5% non-response rate. Finally, the minimum sample size of 383 was obtained. Hence, only 392 HIV positive mothers with children aged 7–20 months were registered in the PMTCT clinics. All eligible mother-child pairs who attended the PMTCT clinics in the selected hospitals during the study period were included consecutively.

### Data collection tools and procedure

A structured interviewer-administered questionnaire was used to collect data from HIV positive mothers with children aged 7–20 months registered in the PMTCT clinics of the selected government hospitals. To maintain consistency, the questionnaire was first translated from English to Amharic (the native language of the study area) and was retranslated to English by professional translators and public health experts. Six clinical nurses and two health officers were assigned as data collectors and supervisors, respectively. Two days’ intensive training was given to data collectors and supervisors on the objective of the study, the confidentiality of information, and techniques for conducting the interview. To address the ethical issues, the data collectors were recruited out of the full-time employees of the respective hospitals. However, such data collectors might be prone to social desirability bias in relation to respondents. So, efforts were made to equip the data collectors with techniques to minimize the bias. Awareness was created among all study participants regarding the objective of the study and on the fact that their response wouldn’t affect the possible treatment they needed. They were also informed that the study was not intended to evaluate their adherence to counseling and treatment given earlier. Moreover, data collectors created a conducive environment by keeping mothers in separate rooms and by making them comfortable during data collection. The tool was pretested on 5% of the total sample out of the study area, at Felegehiwot Referral Hospital to evaluate the acceptability and applicability of the procedures.

### Operational definitions

The dependent variable, prelacteal feeding practice, was measured by asking the mothers whether they gave any food or/and drink to the index child before the establishment of breast milk in the first three days of delivery [3, 24, 36]. Accordingly, if the mother gave any drink or/and food other than breast milk to the child within the first three days of delivery, she was considered to be practicing prelacteal feeding. Mothers’ comprehensive knowledge of infant and young child feeding was assessed using the key components of appropriate feeding practices such as knowledge on the benefits of breastfeeding, time of breastfeeding initiation, colostrum feeding, negative health outcomes of prelacteal feeding, and for how long for an infant should be exclusively breastfeeding, complementary feeding, type of food, time to start complementary feeding, and how to feed a child. Principal component analysis (PCA) was done to compute the composite infant and young child feeding (IYCF) index and converted to the lowest, medium, and a highest terciles. Similarly, household wealth status of the respondents was measured using a composite indicator for urban and rural residents by considering their household assets and properties, and categorizing them into lowest, middle, and highest terciles [42].

### Data processing and analysis

Data were entered into Epi-info version 3.5.3 and exported to the Statistical Package for Social Sciences (SPSS) version 20 for analysis. Descriptive statistics, including frequencies and proportions, were computed using the binary logistic regression model in order to summarize the variables. Variables with a *p*-value of less than 0.2 in the bi-variable analysis were entered into the multivariable analysis. Both Crude Odds Ratio (COR) and Adjusted Odds Ratio (AOR) with 95% confidence intervals were estimated to show the strength of associations. Finally, a *p*-value of less than 0.05 at the multivariable logistic regression analysis was used to identify variables significantly associated with prelacteal feeding practices.

## Results

### Socio-demographic and economic characteristics

Out of the total 392 study participants, 367 HIV positive mothers attending public hospitals were included in the study. The mean (standard deviation) age of the mothers was 31 (±4.4) years. The majority (80.7 and 82.3%, respectively) of the respondents were Orthodox Christians and 20–35 years old. Nearly one-third (30%) of the mothers had no formal education. About 74.9% were married at the moment, and 86.9% of the fathers had primary and above educational status. Almost half (52.6 and 50.4%, respectively) of the children were male, aged 7–12 months (Table 1).

**Table 1:** Socio-demographic and economic characteristics of HIV positive mothers with children aged 7–20 months in government hospitals of North Gondar zone, Northwest Ethiopia, 2016

Variables	Frequency (n)	Percentage (%)
Sex of the child (*n* = 367)		
Female	174	47.4
Male	193	52.6
Age of the child (*n* = 367)		
7-12 months	185	50.4
13-20 months	182	49.5
Mother’s age (*n* = 367)		
20-35 years	302	82.3
> 35 years	65	17.7
Religion (*n* = 367)		
Orthodox	296	80.7
Protestant	52	14.2
Muslim	19	5.1
Mother’s ethnicity (*n* = 367)		
Amhara	286	77.9
Kemant	62	16.9
Tigre	12	3.3
Oromo	7	1.9
Mother’s education (*n* = 367)		
No formal education	110	30
Primary	115	31.3
Secondary	64	17.4
Certificate	18	4.9
College and above	60	16.3
Father’s education (*n* = 367)		
No formal education	48	13.1
Primary	115	31.3
Secondary	87	23.7
Certificate	15	4.1
College and above	102	27.8
Mother’s occupation (*n* = 367)		
Housewife	166	45.2
Government employed	43	11.7
Private employed	53	14.4
Farmer	12	3.3
Merchant	51	13.9
Daily laborer	42	11.4
Father’s occupation (*n* = 367)		
Governmental	116	31.6
Private	33	9.0
Farmer	63	17.2
Merchant	107	29.2
Daily laborer	48	13.1
Mother’s marital status (*n* = 367)		
Married	275	74.9
Currently unmarried	92	25.1
Wealth status (*n* = 367)		
Poor	122	33.2
Medium	130	35.4
Rich	115	31.3

### Maternal and child health service utilization

About 82.6% of the mothers made at least one antenatal care (ANC) visit; 85.6% delivered at health institutions. Out of total 47.4% mothers who reported obstetric problems, the majority encountered prolonged labor. The majority (89.5%) of the respondents received counseling on breastfeeding, and nearly one-third (30.8%) had optimal comprehensive knowledge on infant feeding (Table 2).

**Table 2:** Maternal and child service utilization among HIV positive mothers with children aged 7–20 months in government hospitals of North Gondar zone, Northwest Ethiopia, 2016

Variables	Frequency (n)	Percentage (%)
Antenatal care (ANC) (*n* = 367)		
Yes	303	82.6
No	64	17.4
Number of ANC visit (*n* = 303)		
1	41	13.5
2-3	62	20.5
> 4	200	66.0
Place of delivery (*n* = 367)		
Home	53	14.4
Health institution	314	85.6
Counseling on breastfeeding (*n* = 354)		
Yes	317	89.5
No	37	10.5
Mode of delivery (*n* = 367)		
Spontaneous vaginal delivery	174	47.4
Episotomy	101	27.5
Instrumental delivery	13	3.5
Cesarean section (CS)	79	21.5
Parity (*n* = 367)		
1-3 children	325	88.6
4-6 children	42	11.4
Comprehensive knowledge on IYCF^a^ (*n* = 367)		
Poor	143	39
Medium	113	30.8
Good	111	30.2
Obstetric problem (*n* = 367)		
Yes	174	47.4
No	193	52.6
Type of obstetric problem (*n* = 174)		
Prolonged labor	138	79.3
APH	14	8.0
PPH	20	11.6
Others	2	1.1
Birth weight (n = 333)		
Low birth weight (<2.5kg^b^)	21	6.3
Normal	312	93.7

^a^Infant and Young Child Feeding, ^b^kilogram

### Prevalence of prelacteal feeding

In this study, about 19.1% (95% CI: 0.15, 0.23) of the mothers offered prelacteal feeds to their newborn. The most common prelacteal food given to children was sugar solution (35.7%), plain water (27.1%) and abesh (15.7%). Culture and mothers’ breast problems were the most reported reasons for practicing prelacteal feeding. The majority (79.6%) of the children had the first milk (colostrum) within an hour of delivery (Table 3).

**Table 3:** Feeding practice of children aged 7–20 months born from HIV positive mothers in government hospitals of North Gondar Zone, Northwest Ethiopia, 2016

Variables	Frequency (*n*)	Percentage (%)
Pre-lacteal feeding practice for index child		
Yes	70	19.1
No	297	80.9
Pre-lacteal foods (*n* = 70)		
Butter	8	11.4
plain water	19	27.1
Abesh^a^	11	15.7
Sugar solution	25	35.7
Traditional medicine	7	1.1
Reasons for pre-lacteal feeding (*n* = 70)		
Culture	42	60.0
Breast problem	16	22.8
Others	12	17.2
Colostrum feeding		
Yes	292	79.6
No	75	20.4
Reasons for not feeding colostrum (*n* = 75)		
Not clean	21	28.0
Not comfortable for the baby	11	14.7
Not important	17	22.6
Have breast problem	26	34.7
Have breast illness during breastfeeding (*n* = 363)		
Yes	45	12.4
No	318	87.6

^a^made from roasted and powdered fenugreek diluted with water

### Factors associated with prelacteal feeding

In the bivariable logistic regression analysis, residence, father’s education, wealth status, antenatal care visit, counseling about infant feeding, place of birth, mother’s comprehensive knowledge of infant and young child feeding (IYCF), obstetric problem, and colostrum feeding were factors associated with prelacteal feeding at a *p*-value of less than 0.2. Consequently, these variables were entered into the multivariate logistic regression analysis, and it was noted that fathers’ education, mothers’ antenatal care visits, their counseling on infant feeding and comprehensive knowledge of IYCF, and giving the colostrum were significantly and independently associated with a prelacteal feeding practice.

The odds of prelacteal feeding were higher among illiterate fathers compared with the literate ones (AOR: 5.85; 95% CI: 2.02, 16.92). Mothers who had just one antenatal care visit also had high odds of prelacteal feeding (AOR: 0.07; 95% CI: 0.03, 0.15). Similarly, the odds of prelacteal feeding were high among mothers who did not get counseling about infant feeding (AOR: 3.36; 95% CI: 1. 27, 8.85). Mothers who had medium comprehensive knowledge on IYCF were 62% less likely to practice prelacteal feeding compared with mothers who had poor comprehensive knowledge on IYCF (AOR:0.38; 95% CI: 0.16, 0.88). Mothers who had optimal (good) comprehensive knowledge were consistently 90% less likely to practice prelacteal feeding compared with mothers who had poor comprehensive knowledge (AOR:0.10; 95% CI: 0.03, 0.30). Furthermore, the odds of prelacteal feeding were higher among mothers who discarded the colostrum (AOR: 5.16; 95% CI: 2.32, 11.45) (Table 4).

**Table 4:** Factors associated with prelacteal feeding practice among HIV positive mothers with children aged 7–20 months in government hospitals of North Gondar zone, Northwest Ethiopia, 2016

Variables	Prelacteal feeding		Crude odds ratio	Adjusted odds ratio
	Yes *n* (%)	No *n* (%)	(95% CI)	(95% CI)
Place of residence				
Urban	46 (16.1)	239 (83.9)	0.47 (0.26,0.82)	0.82 (0.23,2.88)
Rural	24 (29.3)	58 (70.7)	1	1
Father’s education				
No formal education	17 (35.4)	31 (64.6)	2.75 (1.42,5.33)	5.85 (2.02,16.92)*
Primary and above	53 (16.6)	266 (83.4)	1	1
Wealth status				
Poor	31 (25.4)	91 (74.6)	1	1
Medium	16 (12.3)	114 (87.7)	0.41 (0.21,0.80)	0.57 (0.20,1.62)
Rich	23 (20.0)	92 (80.0)	0.73 (0.39,1.35)	1.16 (0.44,3.07)
ANC				
Yes	31 (10.2)	272 (89.8)	0.073 (0.039,0.136)	0.07 (0.03,0.15)*
No	39 (60.9)	25 (39.1)	1	1
Counseling about infant feeding				
Yes	49 (15.5)	268 (84.5)	1	1
No	14 (37.8)	23 (62.2)	3.32 (1.60,6.91)	3.36 (1.27,8.85)*
Place of birth				
Home	21 (39.6)	32 (60.4)	3.55 (1.89,6.65)	2.00 (0.79,5.04)
Health institution	49 (15.6)	265 (84.4)	1	1
Comprehensive knowledge on IYCF^a^				
Poor	36 (25.2)	107 (74.8)	1	1
Medium	26 (23.0)	87 (77.0)	0.89 (0.49,1.58)	0.38 (0.16,0.88)*
Good	8 (7.2)	103 (92.8)	0.23 (0.10,0.52)	0.10 (0.03,0.30)*
Have obstetric problem				
Yes	43 (24.7)	131 (75.3)	2.01 (1.18,3.43)	1.72 (0.85,3.47)
No	27 (14.0)	166 (86.0)	1	1
Give colostrum				
Yes	37 (12.7)	254 (87.3)	1	1
No	33 (43.4)	43 (56.6)	5.27 (2.98,9.31)	5.16 (2.32,11.45)*

^*^Variables with *P*-value of < 0.05, ^a^Infant and Young Child Feeding

## Discussion

Infant and young child optimal breastfeeding is essential for survival, growth, and cognitive development; however, inappropriate feeding practices, including prelacteal feeding, remain a problem [4, 43].

This study showed that the prevalence of prelacteal feeding was 19.1% (95% CI: 15%, 23%). The finding was in line with those of studies conducted in different regions of Ethiopia, for instance, in Southern (16.8%) [38], Oromiya (21.9%), and Benshangul-Gumuz regions (23.4%) [36]. However, the prevalence was higher than the reports of other developing countries, such as Malawi (5.4%) [44], Zambia (8.9%) [28]. This might be related to the socio-cultural differences of the study settings. Studies also claimed that mothers’ perceived insufficient and delayed milk production is a common reason for offering prelacteal feeds [45, 46].

However, the finding of this study was lower than the national report of 27%, and that of the Amhara (47.4%) and Somali regions (72.5%) [36]. Like the reports of other studies [47, 48], better care provided to HIV positive mothers might reduce prelacteal feeding and increase appropriate feeding practices. Moreover, the difference of scope between the EDHS report and this study might have contributed to the differences in prelacteal feeding practices. Similarly, the reported low rate of prelacteal feeding in this study compared to the study conducted in Raya Kobo district, north-east Ethiopia, may be due to differences in the participants of the study. For instance, the respondents of the Raya Kobo study were rural dwellers who had poor access to maternal and child health services [33]. The prevalence of prelacteal feeding was high in countries that have better economic status and more educated mothers, like Vietnam (73.3%) [34], Egypt (58%) [49], Thailand (34.6%) [50], Kuwait (81.8%) [51], Bangladesh (92%) [52], and the Philippines (55%) [53]. This might be due to the fact that better wealth status enables mothers to purchase expensive formulae feeds for their newborn before the establishment of breastfeeding [24, 49]. In addition, educated mothers’ unfavorable attitude toward prelacteal feeding, like perceived risks of hypoglycemia and dehydration plus social norms that favor the practice might be the other causes [45, 46].

Out of the variables which showed a significant association with prelacteal feeding at *p*-value of less than 0.05, higher odds of prelacteal feeding were noted among fathers who had no formal education, who didn’t get counseling about infant feeding, and who discarded the colostrum. In contrast, the odds of prelacteal feeding were lower among mothers who attended antenatal care centers and among mothers who had medium and good comprehensive knowledge on IYCF.

In this report, high odds of prelacteal feeding were noted among mothers who discarded the colostrum. This finding is supported by other findings reported in northeast Ethiopia [2], and Bangladesh [54]. This might be due to maternal misperception of the benefits of breastfeeding, especially mothers’ negative attitude on the benefits of the colostrum [41]; breast problems after delivery might also lead to prelacteal feeding. Other reports stated that mothers’ misconception that the colostrum is not important for the child and that it rather makes the newborn sick might be the reasons for discarding the substance [23, 34]. Besides, the introduction of other foods to the newborn before breast milk establishment decreases infant suckling activity and the secretion of milk, subsequently leading to further prelacteal feeds [8, 41].

The odds of prelacteal feeding practices among HIV positive mothers who had medium and good comprehensive knowledge of IYCF were lower compared to their counterparts’. This might be due to the fact that mother’s knowledge of IYCF is an important component of optimal and appropriate child feeding practice. This finding is similar with that of studies conducted elsewhere [34, 39, 55].

Like studies conducted elsewhere [56, 57], high odds of prelacteal feeding practices were noted among illiterate fathers compared to fathers with some level of education. This can be due to the role of education as an important social determinant of health for children. As fathers are educated, their involvement in child feeding practices increases and might have its own impact in practicing appropriate child breastfeeding. Besides, in countries like Ethiopia, where husbands are more influential, any father support in breastfeeding practice enables the mother to avoid prelacteal feeds.

Consistent with previous studies [2, 29, 31, 32], the odds of prelacteal feeding among mothers who had antenatal care visits and counseling on breastfeeding were lower compared with their counterparts. This might be due to the fact that behavioral change communication strategies, such as individual and group counseling, increase appropriate and optimal breastfeeding practices. In addition, breastfeeding counseling during critical periods of pregnancy, including antenatal care visits discourages prelacteal feeding and promotes appropriate and optimal child feeding practices. That is because mothers who have prenatal care visits or maternal and child health services get better access and exposure to health information from health care providers and educational materials that might lead to avoiding prelacteal feeding practices.

The study attempted to show what prelacteal feeding looks like in rural areas, particularly among HIV positive mothers. However, it was not free from limitations, such as inadequate sample size, and inability to include qualitative study methods. In addition, recall and social desirability bias are also possible limitations of the study.

## Conclusion

In this study, the prevalence of prelacteal feeding was high and fathers’ education, colostrum feeding, infant feeding counseling, ANC visits, and IYCF knowledge were significantly associated with it. As a result, strengthening maternal health care utilization, breastfeeding counseling, and IYCF are essential to address the burden.

## Abbreviations

AOR: Adjusted Odds ratio
AIDS: Acquired immune deficiency syndrome
ANC: Antenatal care
ART: Antiretroviral treatment
CI: Confidence interval
EDHS: Ethiopia demographic and health survey
HIV: Human Immune deficiency Virus
IYCF: Infant young child feeding
PCA: Principal component analysis
PMTCT: Prevention of mother to child transmission
SPSS: Statistical package for social sciences
UNICEF: United Nations Children’s Fund
WHO: World Health Organization
